# Symptoms of pre-treatment anxiety are associated with the development of chronic peripheral neuropathy among colorectal cancer patients

**DOI:** 10.1007/s00520-022-06971-1

**Published:** 2022-03-18

**Authors:** Cynthia S. Bonhof, Daniëlle L. van de Graaf, Dareczka K. Wasowicz, Gerard Vreugdenhil, Floortje Mols

**Affiliations:** 1 CoRPS - Center of Research On Psychological Disorders and Somatic Diseases, Department of Medical and Clinical Psychology, Tilburg University, PO Box 90153, 5000 LE Tilburg, The Netherlands; 2 Department of Research, Netherlands Comprehensive Cancer Organisation (IKNL), Utrecht, The Netherlands; 3 Department of Surgery, Elisabeth-TweeSteden Hospital, Tilburg, The Netherlands; 4 Department of Internal Medicine, Máxima Medical Centre, Eindhoven and Veldhoven, The Netherlands

**Keywords:** Colorectal cancer, Peripheral neuropathy, Anxiety symptoms, Depressive symptoms, PROFILES

## Abstract

**Purpose:**

Identifying potentially modifiable predictors of chronic (chemotherapy-induced) peripheral neuropathy (PN) is important, especially in light of the limited treatment options. We aimed to examine pre-treatment anxiety and depressive symptoms as predictors of chronic PN symptom severity in colorectal cancer (CRC) patients up to 2 years after diagnosis.

**Methods:**

Newly diagnosed CRC patients from four Dutch hospitals were eligible for participation. Patients (*N* = 336) completed a questionnaire on anxiety and depressive symptoms (HADS) and sensory (SPN) and motor peripheral neuropathy (MPN) (EORTC QLQ-CIPN20) before initial treatment (baseline) and 1 and 2 years after diagnosis. Patients were included in the analyses if they either developed some level of SPN or MPN symptoms, or experienced a worsening of pre-treatment SPN or MPN symptoms.

**Results:**

At 1-year follow-up, 115 patients (34%) reported SPN symptoms and 134 patients (40%) reported MPN symptoms. Of these patients, SPN and MPN symptoms had not returned to baseline level at 2-year follow-up in, respectively, 51% and 54% of patients. In multivariable regression analyses, neither pre-treatment anxiety symptoms nor pre-treatment depressive symptoms were associated with SPN or MPN symptom severity at 1-year follow-up. At 2-year follow-up, pre-treatment anxiety symptoms (*β* = 0.44, *p* = 0.01), but not depressive symptoms, were associated with SPN symptom severity.

**Conclusions:**

Pre-treatment anxiety symptoms, but not depressive symptoms, were associated with SPN symptom severity 2 years after diagnosis. Future studies are needed that assess whether interventions targeted to reduce anxiety before and during treatment can reduce chronic PN severity or even prevent the persistence of PN.

## Introduction

Peripheral neuropathy (PN) is a common problem in cancer patients. In colorectal cancer (CRC), PN is most often caused by the administration of chemotherapy, specifically oxaliplatin. This oxaliplatin-induced PN mainly results in sensory peripheral neuropathy (SPN), with symptoms such as tingling, numbness, and pain in the hands or feet in a stocking-glove distribution, and motor peripheral neuropathy (MPN), with symptoms such as distal weakness, cramps, gait and balance disturbances, and impaired movements [1, 2]. Chemotherapy-induced PN can resolve after chemotherapy, but becomes a chronic condition in approximately 30% of cancer patients, persisting for 6 months or longer after cessation of chemotherapy [2]. While PN most often results from treatment with a chemotherapeutic agent, it has also been found to be present among cancer patients who did not receive chemotherapy [3], implicating that the disease itself may play a role in the development of PN. Unfortunately, treatment options for PN are limited [4], but much needed, as PN has been shown to severely impact health-related quality of life (HRQoL) [5].

Previous studies on possible predictors for (chemotherapy-induced) PN mostly focused on acute PN, while a limited number of predictors have been investigated for chronic PN. Older age [6], maximum grade of PN during treatment [6], cumulative dose [7], and obesity [8] were found to be related to chronic (chemotherapy-induced) PN, but few studies focused on psychosocial predictors while these predictors play an important role in other chronic pain-related syndromes [9, 10]. According to biopsychosocial models of chronic pain [10], patient’s symptom interpretation will, over time, impact coping styles and behavior patterns such as catastrophizing and repetitious avoidance of activities that may in fact result in the exacerbation of symptoms. Patients who already have a pre-existing vulnerability to develop such non-adaptive coping styles, like those with anxiety and depression, may be at particular risk [11, 12]. Indeed, meta-analyses show that both anxiety and depression are related to chronic pain-related conditions, such as chronic postsurgical pain [13, 14].

Only a small number of studies have been conducted on the association between (chronic) PN and anxiety and depression. While these studies did find a positive association between PN and anxiety and depression [8, 15–19], only two studies included a pre-treatment assessment of anxiety and depression to explore it as a potential risk factor for (chronic) PN [17, 19]. A study among women treated with chemotherapy for breast cancer found that neither anxiety nor depression was associated with chemotherapy-induced PN during active treatment, while pre-treatment anxiety, but not depression, was associated with chemotherapy-induced PN 8 months after completion of chemotherapy [17]. In another study among women with breast cancer, the symptom cluster fatigue, anxiety, and depression was the strongest pre-chemotherapy predictor of numbness and tingling 6 weeks after chemotherapy [19].

Especially in light of the limited treatment options for PN, identifying potentially modifiable predictors of chronic PN is important to help find new treatment opportunities. Therefore, the aim of this prospective study among CRC patients is to examine pre-treatment anxiety and depressive symptoms as predictors of chronic PN symptom severity at 1 and 2 years after diagnosis. As both anxiety and depression have been found to be related to chronic pain-related conditions [13, 14], we hypothesize that both pre-treatment anxiety and depressive symptoms will be associated with PN symptom severity at 1 and 2 years after diagnosis in our sample of CRC patients.

## Methods

### Setting and participants

This study is based on data from the PROCORE study. This prospective, population-based study aimed to examine the longitudinal impact of CRC and its treatment on patient-reported outcomes. Details of the data collection have previously been described elsewhere [20]. In brief, data was collected through PROFILES, which is a registry for the physical and psychosocial impact of cancer and its treatment [21]. PROFILES is directly linked to the Netherlands Cancer Registry (NCR), which collects data from all newly diagnosed cancer patients in the Netherlands [22].

Patients were recruited from four hospitals in the south of the Netherlands: Elisabeth-TweeSteden Hospital, Catharina Hospital, Elkerliek Hospital, and Máxima Medical Centre. All eligible patients newly diagnosed with CRC as a primary tumor between January 2016 and January 2019 were invited to participate. Those previously diagnosed with a different carcinoma, except for basal cell carcinoma of the skin, those with cognitive impairment, and those unable to read or write Dutch, were excluded. All eligible patients were included shortly after diagnosis, before the start of initial treatment. However, in practice, some patients who were previously diagnosed with cancer and those who already started treatment were also included. Therefore, parallel to previous publications based on the PROCORE dataset [23], patients were excluded for analysis if (1) they were previously diagnosed with cancer and reported baseline EORTC QLQ-CIPN20 scores > 0, or (2) they already started chemotherapy.

### Data collection

Eligible patients received an information package about the study from their nurse or case manager. This information package included an information letter, informed consent form, and baseline questionnaire. Follow-up questionnaires were sent at 4 weeks after surgery (when applicable), 1 year after diagnosis, and 2 years after diagnosis. For the current study, the questionnaire that was sent 4 weeks after surgery was not included in the analyses, as this questionnaire does not contain a questionnaire on PN. The PROCORE study was approved by the certified Medical Ethic Committee of Medical research Ethics Committees United (registration number: NL51119.060.14).

### Sociodemographic and clinical characteristics

Patients’ sociodemographic (i.e., age, sex) and clinical (i.e., cancer type, clinical stage, treatment) information was available from the NCR [22]. Comorbidity was assessed with the adapted Self-administered Comorbidity Questionnaire (SCQ) [24]. Questions on partner status and educational level were added to the questionnaire.

### Peripheral neuropathy

The sensory and motor scale of the EORTC QLQ-CIPN20 were used to assess chronic SPN and MPN symptom severity [25]. The items of this questionnaire assess the extent in which the SPN and MPN symptoms were experienced during the past week. Each item is measured on a Likert scale ranging from (1) not at all to (4) very much. Scores were transformed to a 0–100 scale, with higher scores representing higher symptom severity [26]. As the item on hearing problems has been found unlikely to accurately identify PN [27], we calculated the SPN scale excluding this item [28].

### Anxiety and depressive symptoms

Self-reported pre-treatment anxiety and depressive symptoms were measured using the Hospital Anxiety and Depression Scale (HADS) [29]. The HADS consists of 14 items assessing anxiety and depressive symptoms in the last week. Items are answered on a four-point Likert scale. Total scores for both the anxiety and depressive symptom scale range from 0 to 21 with higher scores representing more anxiety and depressive symptoms. A clinically relevant level of anxiety or depression is indicated by a cutoff value of ≥ 8 [30].

### Statistical analyses

NCR data on sociodemographic and clinical characteristics enabled us to compare eligible patients and respondents, using *t*-tests for continuous variables and chi-square (or Fisher’s exact) tests for categorical variables. We also compared differences in sociodemographic and clinical characteristics between (1) patients with SPN and those without SPN at 1-year follow-up, and (2) patients with MPN and those without MPN at 1-year follow-up. Patients were considered as having SPN if they either developed SPN symptoms (i.e., EORTC QLQ-CIPN20 SPN subscale score = 0 at baseline and > 0 at 1-year follow-up) or experienced a worsening of their existing SPN symptoms at 1-year follow-up (i.e., > 0 difference between EORTC QLQ-CIPN20 SPN subscale score at baseline and 1-year follow-up). For MPN, the MPN subscale of the EORTC QLQ-CIPN20 was used. At 1-year follow-up, chemotherapy was completed 7.5 months (range 4–12) prior.

To gain a better understanding of the experienced SPN and MPN symptoms, frequency distributions were calculated in the sample of patients who either developed SPN (or MPN) symptoms or experienced a worsening of their existing SPN (or MPN) at 1-year follow-up. Frequency distributions were also calculated for the subsample of patients whose SPN (or MPN) symptoms had not returned to baseline level at 2-year follow-up.

Finally, to assess the association between pre-treatment anxiety and depressive symptoms (independent variables) and SPN and MPN symptom severity at 1 and 2-year follow-up (dependent variables), hierarchical regression analyses were conducted. In the first step of the regression analyses, pre-treatment anxiety (or depressive) symptoms were included, as well as a priori determined sociodemographic (i.e., age, sex, educational level (high vs. low/medium)) and clinical confounding variables (i.e., tumor type (colon vs. rectum(sigmoid)), cancer stage (III/IV vs. I/II), radiotherapy, oxaliplatin, capecitabine, and pre-treatment SPN/MPN score). In a second step, pre-treatment depressive symptoms (when pre-treatment anxiety symptoms were included in step 1) or pre-treatment anxiety symptoms (when pre-treatment depressive symptoms were included in step 1) were also included in the model.

All analyses were performed using SPSS (IBM SPSS Statistics for Windows, version 24.0 Armonk, NY: IBM Corps USA). A *p* value < 0.05 was considered statistically significant.

## Results

### Patient characteristics

Of the 713 CRC patients who were invited for the study, 68% (*n* = 483) completed the questionnaire at baseline, 52% (*n* = 374) at 1-year follow-up, and 49% (*n* = 347) at 2-year follow-up. A full flow chart of the study has previously been published [20]. Compared with all patients eligible for participation, respondents were younger, more often male, more likely to receive chemotherapy, and less likely to undergo surgery. In addition, they were less often diagnosed with rectosigmoid cancer; they more often had stage III cancer and less often stage IV cancer (data not shown). Of the 374 patients who completed the baseline and 1-year follow-up questionnaires, 32 patients were excluded as they were previously diagnosed with cancer and reported baseline EORTC QLQ-CIPN20 scores > 0 and/or had already started chemotherapy at time of baseline. In addition, six patients had missing data on the EORTC QLQ-CIPN20 and were thus excluded.

Of the remaining 336 patients, 115 patients (34%) developed SPN symptoms or experienced a worsening of existing SPN symptoms at 1-year follow-up (Table 1). Patients with SPN symptoms at 1-year follow-up were on average younger, less often had stage I or II cancer, and more often had stage III cancer compared with patients without SPN symptoms. Additionally, they more often received chemotherapy and specifically oxaliplatin. Regarding MPN, 134 patients (40%) developed MPN symptoms or experienced a worsening of existing MPN symptoms at 1-year follow-up (Table 1). These patients were on average younger, more often female, more often had osteoarthritis, and more often received chemotherapy and specifically oxaliplatin compared with patients without MPN symptoms. In addition, they reported more pre-treatment anxiety and depressive symptoms.

**Table 1:** Baseline sociodemographic and clinical characteristics of colorectal cancer patients stratified by the presence of sensory and motor peripheral neuropathy at 1-year follow-up

	SPN (*n* = 115)	No SPN (*n* = 221)	MPN (*n* = 134)	No MPN (*n* = 202)
Age (mean, SD)	65.1 (9.0)*	67.3 (8.5)	65.3 (9.5)*	67.3 (8.0)
Female sex	44 (38%)	85 (39%)	62 (46%)*	67 (33%)
Partner (yes)	95 (83%)	191 (86%)	111 (83%)	175 (87%)
Education level^a^				
Low	13 (11%)	17 (8%)	11 (8%)	19 (10%)
Medium	67 (58%)	142 (65%)	84 (63%)	125 (62%)
High	35 (30%)	60 (27%)	38 (29%)	57 (28%)
Tumor location				
Colon	85 (74%)	157 (71%)	93 (69%)	149 (74%)
Rectum/rectosigmoid	30 (26%)	64 (29%)	41 (31%)	53 (26%)
TNM stage				
I	19 (17%)^‡^	87 (39%)	35 (26%)	71 (35%)
II	22 (19%)	71 (32%)	37 (28%)	56 (28%)
III	70 (61%)	56 (25%)	56 (42%)	70 (35%)
IV	4 (4%)	6 (3%)	6 (5%)	4 (2%)
Unknown	0 (0%)	1 (1%)	0 (0%)	1 (1%)
Tumor differentiation grade				
Well differentiated	0 (0%)	2 (1%)	0 (0%)	2 (1%)
Moderately differentiated	93 (81%)	178 (81%)	115 (86%)	156 (77%)
Poorly differentiated	6 (5%)	11 (5%)	3 (2%)	14 (7%)
Unknown	16 (14%)	30 (14%)	16 (12%)	30 (15%)
Radiotherapy (yes)	21 (18%)	29 (13%)	18 (13%)	32 (16%)
Chemotherapy				
No	51 (44%)^‡^	181 (82%)	83 (62%)^†^	149 (74%)
Capecitabine	10 (9%)	18 (8%)	9 (7%)	19 (9%)
Oxaliplatin	54 (47%)^‡^	22 (10%)	42 (31%)^†^	34 (17%)
Surgery (yes)	112 (97%)	217 (98%)	131 (98%)	198 (98%)
Number of comorbidities				
None	34 (30%)	60 (27%)	33 (25%)	61 (31%)
One	43 (38%)	70 (32%)	47 (35%)	66 (33%)
Two or more	37 (32%)	89 (41%)	54 (40%)	72 (36%)
Comorbidities associated with PN^b^				
Osteoarthritis	26 (23%)	43 (20%)	35 (26%)^†^	34 (17%)
Rheumatoid arthritis	6 (5%)	10 (5%)	7 (5%)	9 (5%)
Diabetes mellitus	13 (11%)	17 (8%)	13 (10%)	17 (9%)
Pre-treatment anxiety symptoms (mean, SD)	5.2 (4.1)	5.1 (3.9)	5.8 (4.3)*	4.7 (3.7)
Pre-treatment depressive symptoms (mean, SD)	4.1 (3.8)	3.5 (3.5)	4.5 (3.9)^†^	3.2 (3.3)

Abbreviations: *SPN*, sensory peripheral neuropathy; *MPN*, motor peripheral neuropathy; *SD*, standard deviation

Variables may deviate from 100% due to rounding off

Significant difference between either patients with SPN and those without SPN at 1-year follow-up, or patients with MPN and those without MPN at 1-year follow-up at *p < 0.05; ^†^*p* < 0.01; ^‡^*p* < 0.001

^a^Education: low (no or primary school); medium (lower general secondary education or vocational training); high (pre-university education, high vocational training, university)

^b^Most frequent comorbidities associated with peripheral neuropathy

### SPN and MPN symptoms

Among patients with SPN, the symptoms that patients experienced the most at 1-year follow-up were tingling fingers or hands (46%), tingling toes or feet (40%), numbness in toes or feet (38%), and numbness in fingers or hands (32%). At 2-year follow-up, SPN symptoms had not returned to baseline level in 59 out of 115 patients (51%; *n* = 18 missing). Patients still most frequently reported numbness in toes or feet (66%), tingling toes or feet (60%), tingling fingers or hands (59%), and numbness in fingers or hands (47%).

Among patients with MPN, the symptoms that patients experienced the most at 1-year follow-up were difficulty opening a jar or bottle because of weakness in hands (62%), difficulty manipulating small objects with fingers (50%), difficulty climbing stairs or getting up out of a chair because of weakness in legs (42%), and cramps in hands (35%). At 2-year follow-up, MPN symptoms had not returned to baseline in 72 out of 134 patients (54%; *n* = 18 missing). Patients most frequently reported difficulty opening a jar or bottle because of weakness in hands (65%), difficulty manipulating small objects with fingers (50%), cramps in feet (42%), difficulty climbing stairs or getting up out of a chair because of weakness in legs (36%), and cramps in hand (36%).

### Pre-treatment anxiety and depressive symptoms as predictors of PN symptom severity: 1-year follow-up

At 1-year follow-up, both pre-treatment anxiety (*β* = 0.22, *p* = 0.009) (Table 2A) and depressive (*β* = 0.20, *p* = 0.02) (Table 3A) symptoms were significantly associated with worse SPN symptom severity in step 1. Explained variance was 4% and 3% for pre-treatment anxiety and depressive symptoms, respectively. In the second model, in which both predictors were included, neither pre-treatment anxiety nor depressive symptoms were significantly associated with SPN symptom severity. In contrast, younger age, low/medium educational level, colon cancer, treatment with oxaliplatin, and worse pre-treatment SPN score were significantly associated with worse SPN severity.

**Table 2:** Association between pre-treatment anxiety symptoms and SPN and MPN symptom severity at 1-year (A) and 2-year (B) follow-up among CRC patients with chronic peripheral neuropathy

	A. 1-year follow-up				B. 2-year follow-up			
	SPN symptom severity		MPN symptom severity		SPN symptom severity		MPN symptom severity	
	Beta	Adj *R*^2^	Beta	Adj *R*^2^	Beta	Adj *R*^2^	Beta	Adj *R*^2^
Step 1								
Pre-treatment anxiety symptoms	**0.22**^**†**^		0.08		**0.43**^‡^		0.30*	
Age	− 0.18*		− 0.13		− 0.20		− 0.07	
Sex (female)	− 0.03		0.02		0.02		− 0.12	
Educational level (high)	− 0.17		0.03		− 0.11		0.09	
Type tumor (colon)	0.28*		0.03		0.29		− 0.11	
Stage (III and IV)	− 0.23		− 0.15		− 0.38*		− 0.22	
Radiotherapy	− 0.18		− 0.08		0.02		0.03	
Chemotherapy—oxaliplatin	**0.69**^**‡**^		**0.43**^**‡**^		0.61*		0.31	
Chemotherapy—capecitabine	0.15		0.21*		0.25		0.18	
Pre-treatment SPN/MPN score	0.22*		**0.59**^**‡**^		0.18		**0.44**^**†**^	
		0.31^a^		0.33^a^		0.23^a^		0.21^a^
Step 2								
Pre-treatment anxiety symptoms	0.16		0.10		0.44*		0.25	
Age	− 0.19*		− 0.12		− 0.20		− 0.08	
Sex (female)	− 0.03		0.01		0.03		− 0.12	
Educational level (high)	− 0.17*		0.03		− 0.11		0.09	
Type tumor (colon)	0.29*		0.02		0.29		− 0.09	
Stage (III and IV)	− 0.23		− 0.15		− 0.38		0.21	
Radiotherapy	− 0.19		− 0.07		0.02		0.02	
Chemotherapy—oxaliplatin	**0.70**^**‡**^		**0.43**^**‡**^		0.61*		0.31	
Chemotherapy—capecitabine	0.14		0.21*		0.25		0.17	
Pre-treatment SPN/MPN score	**0.22**^**†**^		**0.60**^**‡**^		0.18		**0.34**^**†**^	
Pre-treatment depressive symptoms	0.10		− 0.03		− 0.01		0.06	
		0.31^a^		0.33^a^		0.22^a^		0.20^a^

Abbreviations: *SPN*, sensory peripheral neuropathy; *MPN*, motor peripheral neuropathy; *CRC*, colorectal cancer; *Adj*, adjusted. *Significant at *p* < 0.05; ^†^significant at *p* < 0.01; ^‡^significant at *p* < 0.001. ^a^Change in adjusted *R*^2^ is significant

**Table 3:** Association between pre-treatment depressive symptoms and SPN and MPN symptom severity at 1-year (A) and 2-year (B) follow-up among CRC patients with chronic peripheral neuropathy

	A. 1-year follow-up				B. 2-year follow-up			
	SPN symptom severity		MPN symptom severity		SPN symptom severity		MPN symptom severity	
	Beta	Adj *R*^2^	Beta	Adj *R*^2^	Beta	Adj *R*^2^	Beta	Adj *R*^2^
Step 1								
Pre-treatment depressive symptoms	0.20*		0.04		0.29*		0.26*	
Age	− 0.21*		− 0.14		− 0.25		− 0.13	
Sex (female)	− 0.03		0.02		0.03		− 0.09	
Educational level (high)	− 0.18*		0.03		− 0.12		0.07	
Type tumor (colon)	0.29*		0.04		0.34		− 0.08	
Stage (III and IV)	− 0.22		− 0.15		− 0.29		− 0.18	
Radiotherapy	− 0.19		− 0.08		− 0.07		− 0.02	
Chemotherapy—oxaliplatin	**0.69**^**‡**^		**0.43**^**‡**^		0.52*		0.31	
Chemotherapy—capecitabine	0.13		0.20*		0.16		0.13	
Pre-treatment SPN/MPN score	**0.24**^**†**^		**0.59**^**‡**^		0.15		**0.42**^**†**^	
		0.30^a^		0.38^a^		0.11		0.19^a^
Step 2								
Pre-treatment depressive symptoms	0.10		− 0.03		0.01		0.06	
Age	− 0.19*		− 0.12		− 0.20		− 0.08	
Sex (female)	− 0.03		0.01		0.03		− 0.12	
Educational level (high)	− 0.17*		0.03		− 0.11		0.09	
Type tumor (colon)	0.29*		0.02		0.29		− 0.09	
Stage (III and IV)	− 0.23		− 0.15		− 0.38		0.21	
Radiotherapy	− 0.19		− 0.07		0.02		0.02	
Chemotherapy—oxaliplatin	**0.70**^**‡**^		**0.43**^**‡**^		0.61*		0.31	
Chemotherapy—capecitabine	0.14		0.21*		0.25		0.17	
Pre-treatment SPN/MPN score	**0.22**^**†**^		**0.60**^**‡**^		0.18		**0.34**^**†**^	
Pre-treatment anxiety symptoms	0.16		0.10		0.44*		0.25	
		0.31^a^		0.38^a^		0.22^a^		0.20^a^

Abbreviations: *SPN*, sensory peripheral neuropathy; *MPN*, motor peripheral neuropathy; *CRC*, colorectal cancer; *Adj*, adjusted. *Significant at *p* < 0.05; ^†^significant at *p* < 0.01; ^‡^significant at *p* < 0.001. ^a^Change in adjusted *R*^2^ is significant

Regarding MPN, pre-treatment anxiety (Table 2A) and depressive symptoms (Table 3A) were both not associated with MPN symptom severity at 1-year follow-up in either of the two models. Worse pre-treatment MPN score and treatment with oxaliplatin or capecitabine were associated with worse MPN symptom severity.

### Pre-treatment anxiety and depressive symptoms as predictors of PN symptom severity: 2-year follow-up

At 2-year follow-up, both pre-treatment anxiety (*β* = 0.43, *p* = 0.001) (Table 2B) and depressive (*β* = 0.29, *p* = 0.03) (Table 3B) symptoms were significantly associated with worse SPN symptom severity in step 1. Explained variance was 17% for anxiety symptoms and 8% for depressive symptoms, respectively. In the second model, in which pre-treatment anxiety and depressive symptoms were both included, pre-treatment anxiety (*β* = 0.44, *p* = 0.01) remained significantly associated with SPN symptom severity, explaining 10% of its variance, while depressive symptoms were no longer associated with SPN symptom severity. Treatment with oxaliplatin was also associated with worse SPN symptom severity.

Both pre-treatment anxiety (*β* = 0.30, *p* = 0.01) (Table 2B) and depressive symptoms (*β* = 0.26, *p* = 0.03) (Table 3B) were significantly associated with worse MPN symptom severity in step 1, explaining 8% and 6% of its variance. However, these associations disappeared when both predictors were included in the second model, leaving a worse pre-treatment MPN score as the only significant predictor of MPN symptom severity.

## Discussion

This prospective, population-based cohort study first showed that among CRC patients, 34% developed some level of SPN symptoms or experienced a worsening of their existing SPN symptoms at 1 year after diagnosis. In addition, symptoms did not return to baseline level at 2 years after diagnosis in 51% of these patients. The development or worsening of existing MPN symptoms were reported by 40% of CRC patients, of which 54% still reported MPN levels above baseline level at 2 years after diagnosis. The rates are thus somewhat higher than the reported overall chemotherapy-induced PN prevalence of 30% at 6 months or more after chemotherapy has ended [2]. This may be due to differences in method of PN assessment. In contrast to most of the studies included in the meta-analysis [2], we only used the patient-reported EORTC QLQ-CIPN20 [25], and patient-reported measures are found to be more sensitive in detecting beginning or mild PN than objective or clinician-rated assessments [31]. In addition, in this study, we also included patients with mild PN symptoms, as we included those with any score above 0 on the EORTC QLQ-CIPN20 SPN or MPN subscale.

The main objective of this study was to examine pre-treatment anxiety and depressive symptoms as predictors of chronic PN symptom severity at 1 and 2 years after diagnosis. We expected both pre-treatment anxiety and depressive symptoms to be associated with PN symptom severity at both 1 and 2 years after diagnosis. The results showed that neither pre-treatment anxiety nor depressive symptoms were significantly associated with SPN or MPN symptom severity at 1 year after diagnosis. At 2-year follow-up, pre-treatment anxiety symptoms, but not depressive symptoms, were significantly associated with SPN symptom severity. These results are in line with a study among breast cancer patients, in which neither pre-treatment anxiety nor depression was found to be associated with chemotherapy-induced PN during active treatment, but that pre-treatment anxiety was associated with chemotherapy-induced PN 8 months after completion of chemotherapy [17].

The association between pre-treatment anxiety symptoms and SPN symptom severity may be explained through the biopsychosocial model of chronic pain [10]. According to this widely accepted model, pain is a complex and dynamic interplay between biological, psychological, and social factors. Perceptual and cognitive processes, like catastrophizing, fear-avoidance beliefs, and hypervigilance to negative information, may increase PN symptoms and may even cause acute PN to become chronic. For example, acute PN symptoms may cause patients to catastrophize their PN and perceive certain activities, such as physical exercise, as potentially detrimental for their condition. This generally leads to the avoidance of such activities, which may then actually result in the exacerbation of their PN symptoms, leading to increased anxiety and avoidance behavior. Finally, patients may find themselves in a vicious circle of escalating anxiety, avoidance behavior, and exacerbation of PN symptoms [12]. At particular risk for chronic PN may be those who have a pre-existing vulnerability to develop such non-adaptive psychological reactions. Within the context of the biopsychosocial model, a diathesis-stress framework proposes that individuals have certain inherent vulnerabilities, or diathesis, to develop certain disorders or difficulties when exposed to stressors such as PN [11]. Pre-treatment (latent) anxiety can be activated or exacerbated by the stress of PN and the exacerbated anxiety may then cause the PN to develop into a chronic condition.

In line with the biopsychosocial model of chronic pain, it has been hypothesized that acute chemotherapy-induced PN may primarily be caused by the neurotoxic effects of the chemotherapeutic agent, while chronic chemotherapy-induced PN is more likely to be affected by predisposing psychological factors that maintain the chemotherapy-induced PN symptoms [17]. Chemotherapy-induced PN can be considered chronic when symptoms persist for 6 months or longer after cessation of chemotherapy [2]. In our study, chemotherapy was completed 4 to 12 months prior to 1-year follow-up. Therefore, the PN may not yet have turned into a chronic condition for all patients. This may explain why we did find an association between pre-treatment anxiety and SPN symptom severity at 2-year follow-up, while no such association was found at 1-year follow-up.

An increased production of pro-inflammatory cytokines may also play a role in the persistence of PN among patients with pre-treatment anxiety. Anxiety, as well as (chemotherapy-induced) PN, has been associated with increased pro-inflammatory cytokines, such as IL-6 [32, 33]. As pro-inflammatory cytokines play a crucial role in nerve repair, anxiety might interfere with recovery from the nerve injury in PN.

Interestingly, in our study, pre-treatment anxiety symptoms were not associated with MPN symptom severity at 2-year follow-up, but neither was treatment with the chemotherapeutic agents capecitabine or oxaliplatin. Treatment with these chemotherapeutic agents was associated with MPN symptom severity at 1-year follow-up, but pre-treatment MPN score was the only significant risk factor for MPN symptom severity at 2-year follow-up. In our previous study among the same study population, we examined the course of PN among both patients who received chemotherapy and those who did not [23]. In both groups, MPN symptoms had increased at 1-year follow-up, with those who received chemotherapy reporting significantly more MPN symptoms. At 2-year follow-up, symptoms declined among this group and while symptoms did not return to baseline level, there was no longer a significant difference with patients who did not receive chemotherapy, as MPN symptoms further increased among this group. It could be that, at 2-year follow-up, MPN symptoms were mostly due to (pre-existing) PN-related comorbidity such as osteoarthritis, diabetes mellitus, and rheumatoid arthritis. We also find it difficult to offer a sensible explanation as to why depressive symptoms were not associated with SPN symptom severity while it has been related to pain intensity in other samples [13], or why pre-treatment anxiety symptoms but not depressive symptoms would be related to SPN symptom severity. Future research aimed to elucidate these findings is necessary.

The findings of our study add to the limited knowledge on potentially modifiable risk factor for chronic PN, especially in light of the limited treatment options. Based on this study, it is too early to make any recommendations for clinical practice. However, as PN negatively impacts the HRQoL of CRC survivors [18, 23, 34], we would recommend future studies to examine whether interventions targeted to reduce anxiety before and during treatment can reduce the severity of chronic PN or even prevent the persistence of PN.

Some limitations should be acknowledged. First, no information was available on chemotherapy dosage, number of cycles, and possible dose reduction, while these factors are important determinants of chemotherapy-induced PN severity [2]. Secondly, it has been advised that self-reported (chemotherapy-induced) PN measures should preferably be combined with clinician-rated neurological assessment tools [35]. Therefore, the use of only the EORTC QLQ-CIPN20 is another limitation. However, due to the subjective nature of PN symptoms and the typical underestimation of PN severity by healthcare professionals [36], we feel that patient-reported assessment of PN is of greater importance. In addition, patients lost to follow-up could have stopped participating because of severe PN symptoms in their hands, which could have impacted our findings. Finally, eligible patients and respondents of this study differed in some sociodemographic and clinical characteristics. Therefore, generalization of our findings should be done with caution.

In conclusion, this study showed that neither pre-treatment anxiety symptoms nor pre-treatment depressive symptoms were associated with SPN or MPN symptom severity 1 year after diagnosis, while pre-treatment anxiety symptoms, but not depressive symptoms, were associated with SPN symptom severity 2 years after diagnosis. These results highlight the importance of future studies that examine the effectiveness of interventions aimed to reduce anxiety before and during treatment in reducing chronic PN severity or even preventing the persistence of PN.

## Data Availability

The data that support the findings of this study are available from the PROFILES registry (www.profilesregistry.nl).

## Abbreviations

CIPN: Chemotherapy-induced peripheral neuropathy
CRC: Colorectal cancer
HRQoL: Health-related quality of life
MPN: Motor peripheral neuropathy
NCR: Netherlands Cancer Registry
PROFILES: Patient Reported Outcomes Following Initial treatment and Long term Evaluation of Survivorship
PN: Peripheral neuropathy
SPN: Sensory peripheral neuropathy
